# 

**Published:** 1890-03

**Authors:** 


### CONCERNING THE TENTH INTERNATIONAL MEDICAL CONGRESS.

110 West 14th Street.

NEW YORK, March 7th, 1890.

*Editor Daniel's Texas Medical Journal:*

I am directed by the Secretary General of the Tenth International Congress to give the greatest possible publicity to the circular, the main points of which I herewith transmit to you with the request that they be published.

Very respectfully yours,

A. JACOBI, M. D.

### INVITATION FOR AN INTERNATIONAL MEDICAL AND SCIENTIFIC EXHIBITION.

In connection with the Tenth International Medical Congress, to be held in Berlin, between the Fourth and Tenth of August, there is to be an International Medical and Scientific Exhibition. The exhibits will be of an exclusively scientific nature, as follows:

New and improved scientific instruments and apparatuses for biological and strictly medical purposes, inclusive of apparatuses for photography and spectral analysis as far as applicable to medicine.

New objects and preparations in pharmacological chemistry and pharmacy.

New foods.

New or improved instruments subservient to any of the departments of medicine, including electrotherapy.

New plans and models of hospitals, convalescent homes, and disinfecting and bathing institutions and apparatuses.

New arrangements for nursing, including transportation, baths, etc.

New apparatuses in hygiene.

Applications or inquiries inscribed "Ausstellungs-Angelegenheit," and accompanied with a printed card containing the name and address of the firm thus applying, ought to be directed to the Secretary General, Dr. O. Lassar, Carlstrasse, No. 19, Berlin, N. W., Germany.

R. VIRCHOW, President.

E. VON BERGMANN, E. LEYDEN, W. WALDEYER, Vice-Presidents.

O. LASSAR, Secretary General.

### TENTH INTERNATIONAL MEDICAL CONGRESS.

(To be held in Berlin, between the fourth and tenth of August, 1890.)

The JOURNAL is in receipt of a letter from the passenger agent of the Netherlands-American Steam Navigation Company, New

York, and publishes the following extracts for the information of those who contemplate attending the Congress:

"Dr. W. B. Atkinson, (the Secretary of the A. M. A.), is Secretary of the American Medical Association's Committee on Transportation. He has made special arrangements with our line for the passage of the members who will attend the Congress this summer. Our line was chosen because we sail directly to the European continent, and our landing ports, Rotterdam, Amsterdam, Boulogne, etc., are so well situated, being in the center of all European points likely to be visited, that from there the principal cities are reached at a minimum expense. Our steamers are large, magnificent passenger boats, provided with all modern improvements, and having such accommodations as to fully insure the comfort of the passengers. For members of the Texas State Medical Association, the rate will be $90 for accommodations classed A; $105 for those AA, per berth and round trip to Amsterdam, Rotterdam or Boulogne, etc. These rates are to be obtained by applying to Dr. Atkinson for a certificate, and they include a free passage to London, via Flushing Queenboro, one way, on the outward trip, for those who desire to visit that city. We reserve the very best located rooms for the members of the Medical Association (provisionally), in the steamers sailing between May 10th, and July 19th, which we hold up to a certain latest date for each departure."

If any of the readers of the JOURNAL intend going to the Congress, they can get all the necessary information as to the time of sailing of this line of steamers, etc., by writing to the General Passenger Agent, at 39 Broadway, N. Y.

### AMERICAN MEDICAL ASSOCIATION.

PHILADELPHIA, 1400 Pine St., S. W. cor. Broad.

The Forty-first Annual Session will be held in Nashville, Tenn., on Tuesday, Wednesday, Tursday and Friday, May 20, 21, 22, and 23, commencing on Tuesday, at 11 a. m.

The delegates shall receive their appointment from permanently organized State Medical Societies, and such County and District Medical Societies as are recognized by representation in their respective State Societies, and from the Medical Department of the Army and Navy, and the Marine-Hospital Service of the United States.

Each State, County and District Medical Society entitled to representation shall have the privilege of sending to the Association one delegate for every ten of its regular resident members, and one for every additional fraction of more than half that number; provided, however, that the number of delegates for any particular State, territory, county, city or town shall not exceed the ratio of one in ten of the resident physicians who may have signed the Code of Ethics of the Association.

Members by Application.—Members by application shall consist of such members of the State, County, and District Medical Societies entitled to representation in this Association, as shall make application in writing to the Treasurer, and accompany said application with a certificate of good standing, signed by the President and Secretary of the Society of which they are members, and the amount of the annual membership fee, five dollars. They shall have their names entered upon the roll, and have all the rights and privileges accorded to permanent members, and shall retain their membership upon the same terms.

The following resolution was adopted at the session of 1888:

"That in future each delegate or permanent member shall, when he registers, also record the name of the Section, if any, that he will attend, and in which he will cast his vote for Section officers."

Addresses: On General Medicine, by Dr. N. S. Davis, Chicago, Ill.; on General Surgery, by Dr. Samuel Logan, New Orleans, La.; on State Medicine, by Dr. Alfred L. Carroll, New York, N. Y.

Committee of Arrangements: Dr. Wm. T. Briggs, Chairman, Nashville, Tenn.

WILLIAM B. ATKINSON, Permanent Secretary.

A CIRCULAR, received as the last pages go to press, signed J. P. Arthur, President, and L. W. Cock, Secretary, [illegible]nnounces that the Laredo Medical District Association will meet in Laredo on the first Monday in April, at 8:30 p. m. to perfect the organization.

